# 1-(4-Hy­droxy­phen­yl)piperazine-1,4-diium tetra­chlorido­cobalt(II) monohydrate

**DOI:** 10.1107/S1600536814001767

**Published:** 2014-01-31

**Authors:** Marwa Mghandef, Habib Boughzala

**Affiliations:** a Laboratoire de Matériaux et Cristallochimie, Faculté des Sciences de Tunis, Université de Tunis El Manar, 2092 Manar II Tunis, Tunisia

## Abstract

The asymmetric unit of the title inorganic–organic hybrid compound, (C_10_H_16_N_2_O)[CoCl_4_]·H_2_O, consists of a tetrahedral [CoCl_4_]^2−^ anion, together with a [C_10_H_18_N_2_O]^2+^ cation and a water mol­ecule. Crystal cohesion is achieved through N—H⋯Cl, O—H⋯Cl and N—H⋯O hydrogen bonds between organic cations, inorganic anions and the water mol­ecules, building up a three-dimensional network.

## Related literature   

For spectroscopic and electrochemical properties of hybrid compounds, see: Bu *et al.* (2001 ▶). For a similar structural arrangement, see: Aza­dbakht *et al.* (2012 ▶). For the coordination of cobalt, see: Reiss (2013 ▶); Oh *et al.* (2011 ▶).
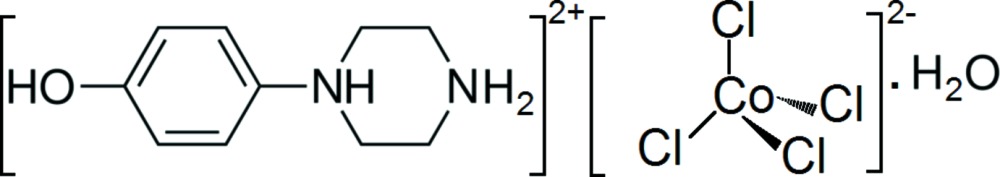


## Experimental   

### 

#### Crystal data  

(C_10_H_16_N_2_O)[CoCl_4_]·H_2_O
*M* *_r_* = 398.99
Triclinic, 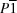

*a* = 7.455 (1) Å
*b* = 8.002 (2) Å
*c* = 14.105 (1) Å
α = 91.72 (1)°
β = 96.98 (1)°
γ = 99.19 (1)°
*V* = 823.4 (2) Å^3^
*Z* = 2
Mo *K*α radiation
μ = 1.69 mm^−1^
*T* = 298 K
0.6 × 0.3 × 0.2 mm

#### Data collection  

Enraf–Nonius CAD-4 diffractometer
Absorption correction: ψ scan (North *et al.*, 1968 ▶) *T*_min_ = 0.607, *T*_max_ = 0.712
4220 measured reflections
3586 independent reflections
3110 reflections with *I* > 2σ(*I*)
*R*_int_ = 0.027
2 standard reflections every 120 min intensity decay: 1%

#### Refinement  

*R*[*F* ^2^ > 2σ(*F* ^2^)] = 0.037
*wR*(*F* ^2^) = 0.102
*S* = 1.08
3586 reflections
245 parameters
All H-atom parameters refined
Δρ_max_ = 0.73 e Å^−3^
Δρ_min_ = −0.45 e Å^−3^

### 

Data collection: *CAD-4 EXPRESS* (Enraf–Nonius, 1994 ▶); cell refinement: *CAD-4 EXPRESS*; data reduction: *XCAD4* (Harms & Wocadlo, 1995 ▶); program(s) used to solve structure: *SHELXS97* (Sheldrick, 2008 ▶); program(s) used to refine structure: *SHELXL97* (Sheldrick, 2008 ▶); molecular graphics: *DIAMOND* (Brandenburg, 2008 ▶); software used to prepare material for publication: *publCIF* (Westrip, 2010 ▶).

## Supplementary Material

Crystal structure: contains datablock(s) I, New_Global_Publ_Block. DOI: 10.1107/S1600536814001767/cq2009sup1.cif

Structure factors: contains datablock(s) I. DOI: 10.1107/S1600536814001767/cq2009Isup2.hkl

CCDC reference:

Additional supporting information:  crystallographic information; 3D view; checkCIF report

## Figures and Tables

**Table 1:** Selected bond lengths (Å)

Co—Cl2	2.2475 (7)
Co—Cl4	2.2772 (7)
Co—Cl1	2.2777 (7)
Co—Cl3	2.2868 (7)

**Table 2:** Hydrogen-bond geometry (Å, °)

*D*—H⋯*A*	*D*—H	H⋯*A*	*D*⋯*A*	*D*—H⋯*A*
O*W*—H*WA*⋯Cl3^i^	0.87 (4)	2.59 (4)	3.381 (3)	150 (4)
O*W*—H*WB*⋯Cl1^ii^	0.78 (4)	2.51 (4)	3.264 (3)	164 (4)
O—H1⋯Cl3^iii^	0.79 (4)	2.41 (4)	3.177 (3)	165 (4)
N1—H1*N*⋯Cl4^iv^	0.94 (3)	2.24 (3)	3.171 (2)	170 (2)
N2—H2*NA*⋯O*W*	0.83 (4)	2.06 (4)	2.807 (2)	151 (3)
N2—H2*NB*⋯Cl1^v^	0.91 (3)	2.47 (3)	3.267 (2)	147 (2)
N2—H2*NB*⋯Cl2^v^	0.91 (3)	2.81 (3)	3.324 (2)	117 (2)

Symmetry codes: (i) 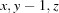; (ii) 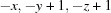; (iii) 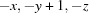; (iv) 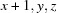; (v) 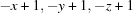.

**Fig. 1.**
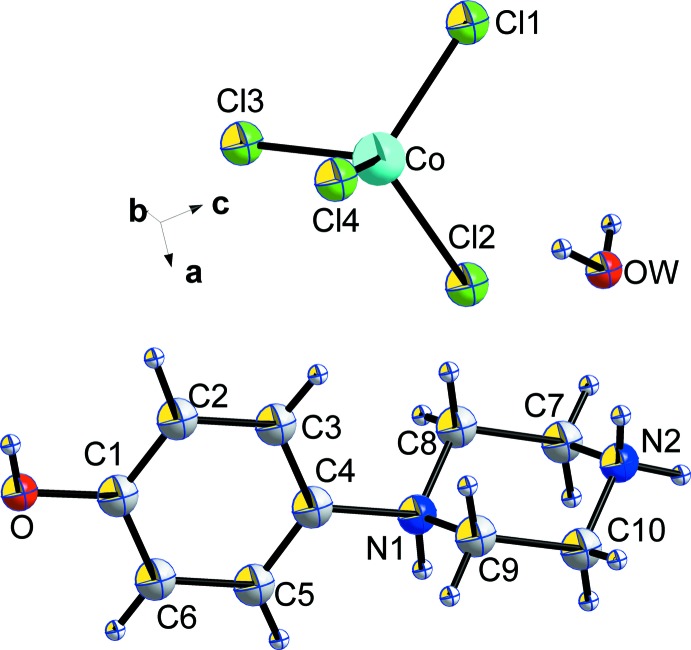
An ORTEP of the molecular entities of [C10H18N2O] [CoCl4]·H2O showing the atom numbering scheme. Displacement ellipsoids are drawn at 50% probability level. Symmetry codes: (i) x, y - 1, z; (ii) -x, -y + 1, -z + 1; (iii) -x, -y + 1, -z; (iv) x + 1, y, z; (v) -x + 1, -y + 1, -z + 1.

**Fig. 2.**
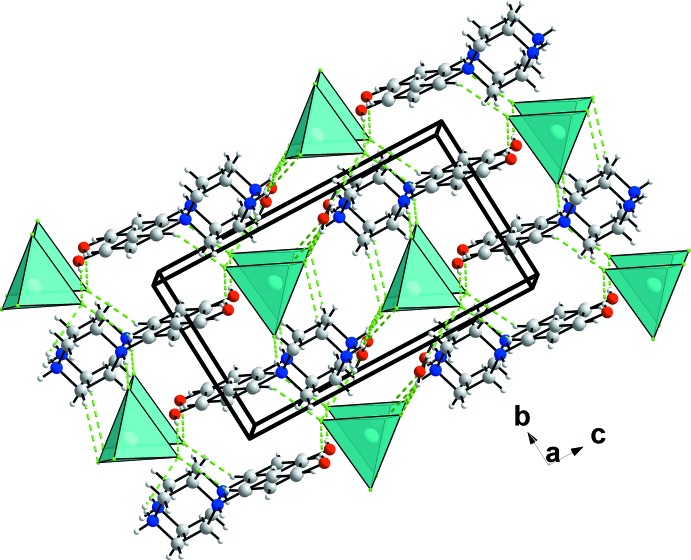
Projection of the [C10H18N2O] [CoCl4]·H2O structure showing the hydrogen bonds as dashed lines and the alternating stacking of organic and inorganic layers along [011].

## supplementary crystallographic information

### 1. Comment

Recently, the synthesis of organic-inorganic hybrid compounds has attracted increasing interest, not only from a structural point of view, but also because of their diverse optical properties and various applications in catalysis, electrical conductivity and photochemistry (Bu *et al.*, 2001).

Here we report the synthesis and structural characterisation of the organic-inorganic hybrid compound, (1-hydroxyphenyl)piperazine-1,4-diium tetrachloridocobalt(II) monohydrate, [C_10_H_18_N_2_O] [CoCl_4_]·H_2_O.

The asymmetric unit of this compound is composed of one tetrachlorocobalt(II) anion, one organic cation and one isolated water molecule, as shown in Figure 1. The coordination geometry of the Co(II) ion is tetrahedral with Co—Cl bond lengths ranging from 2.2475 (7) to 2.2868 (7) Å, as observed in similar compounds (Oh *et al.*; 2011), (Reiss; 2013), (Azadbakht *et al.*; 2012).

The CoCl_4_ groups are isolated (0-D anionic network) and connected to three organic cations by N—H···Cl and O—H···Cl hydrogen bonds and to two water molecules by O—H···Cl hydrogen bonds.

The organic cation, [C_10_H_18_N_2_O]^2+^, contains a piperazindium ring in a chair conformation and a planar aromatic ring (r.m.s. deviation = 0.0119 Å). The angle between the mean planes of the phenyl and piperazindium rings is about 78.0°. The crystal structure can be described as alternating stacking of organic and inorganic layers along [011], as shown in Figure 2. Water molecules link adjacent inorganic sheets.

The stability and the cohesion between the different components of the structure are assured by the water molecules connected to the organic cations through N—H···O hydrogen bonds and to the tetrahedral vertices of the tetrachlorocobalt (II) anions to build up a three-dimensional network.

### 2. Experimental

A mixture of chloride cobalt (II) CoCl_2_·H_2_O (0.24 g) and 1-acetyl-4-(4-hydroxyphenyl)piperazine (C_12_H_16_N_2_O_2_) (0.11 g) (molar ratio 1:1) was dissolved in an aqueous solution of hydrochloric acid. The mixture was stirred then kept at room temperature. Blue crystals of the title compound were obtained two weeks later. The (1-hydroxyphenyl) cations are formed by loss of the acetyl group on acid hydrolysis.

### 3. Refinement

The hydrogen atoms were located in difference Fourier maps. Those attached to carbon were placed in calculated positions (C—H = 0.86 – 1.00 Å) while those attached to nitrogen and oxygen were placed in the experimental positions and their coordinates adjusted to give N—H = 0.83 Å and O—H = 0.81 Å. All were included as riding contributions with isotropic displacement parameters 1.2 - 1.5 times those of the attached atoms.

### Crystal data

**Table:**

(C_10_H_16_N_2_O)[CoCl_4_]·H_2_O	*Z* = 2
*M**_r_* = 398.99	*F*(000) = 406
Triclinic, *P*1	*D*_x_ = 1.609 Mg m^−^^3^
Hall symbol: -P 1	Mo *K*α radiation, λ = 0.71073 Å
*a* = 7.455 (1) Å	Cell parameters from 25 reflections
*b* = 8.002 (2) Å	θ = 10–15°
*c* = 14.105 (1) Å	µ = 1.69 mm^−^^1^
α = 91.72 (1)°	*T* = 298 K
β = 96.98 (1)°	Prism, blue
γ = 99.19 (1)°	0.6 × 0.3 × 0.2 mm
*V* = 823.4 (2) Å^3^	

### Data collection

**Table:**

Enraf–Nonius CAD-4 diffractometer	3110 reflections with *I* > 2σ(*I*)
Radiation source: fine-focus sealed tube	*R*_int_ = 0.027
Graphite monochromator	θ_max_ = 27.0°, θ_min_ = 2.6°
non–profiled ω/2θ scans	*h* = −9→1
Absorption correction: ψ scan (North *et al.*, 1968)	*k* = −10→10
*T*_min_ = 0.607, *T*_max_ = 0.712	*l* = −17→17
4220 measured reflections	2 standard reflections every 120 min
3586 independent reflections	intensity decay: 1%

### Refinement

**Table:**

Refinement on *F*^2^	Secondary atom site location: difference Fourier map
Least-squares matrix: full	Hydrogen site location: inferred from neighbouring sites
*R*[*F*^2^ > 2σ(*F*^2^)] = 0.037	All H-atom parameters refined
*wR*(*F*^2^) = 0.102	*w* = 1/[σ^2^(*F*_o_^2^) + (0.0592*P*)^2^ + 0.2936*P*] where *P* = (*F*_o_^2^ + 2*F*_c_^2^)/3
*S* = 1.08	(Δ/σ)_max_ = 0.001
3586 reflections	Δρ_max_ = 0.73 e Å^−^^3^
245 parameters	Δρ_min_ = −0.45 e Å^−^^3^
0 restraints	Extinction correction: *SHELXL97* (Sheldrick, 2008), Fc^*^=kFc[1+0.001xFc^2^λ^3^/sin(2θ)]^-1/4^
Primary atom site location: structure-invariant direct methods	Extinction coefficient: 0.019 (2)

### Special details

**Table:**

Geometry. All e.s.d.'s (except the e.s.d. in the dihedral angle between two l.s. planes) are estimated using the full covariance matrix. The cell e.s.d.'s are taken into account individually in the estimation of e.s.d.'s in distances, angles and torsion angles; correlations between e.s.d.'s in cell parameters are only used when they are defined by crystal symmetry. An approximate (isotropic) treatment of cell e.s.d.'s is used for estimating e.s.d.'s involving l.s. planes.
Refinement. Refinement of *F*^2^ against ALL reflections. The weighted *R*-factor *wR* and goodness of fit *S* are based on *F*^2^, conventional *R*-factors *R* are based on *F*, with *F* set to zero for negative *F*^2^. The threshold expression of *F*^2^ > σ(*F*^2^) is used only for calculating *R*-factors(gt) *etc*. and is not relevant to the choice of reflections for refinement. *R*-factors based on *F*^2^ are statistically about twice as large as those based on *F*, and *R*- factors based on ALL data will be even larger.

### Fractional atomic coordinates and isotropic or equivalent isotropic displacement parameters (Å^2^)

**Table:**

	*x*	*y*	*z*	*U*_iso_*/*U*_eq_	
Co	0.13891 (4)	0.68672 (4)	0.31676 (2)	0.03430 (13)	
Cl1	−0.02197 (8)	0.72320 (8)	0.44103 (4)	0.04349 (16)	
Cl2	0.43614 (7)	0.72221 (8)	0.37771 (4)	0.04201 (16)	
Cl3	0.09100 (10)	0.89884 (9)	0.21635 (5)	0.05405 (19)	
Cl4	0.04924 (9)	0.43236 (8)	0.23402 (5)	0.0534 (2)	
N1	0.6315 (3)	0.2602 (2)	0.20897 (12)	0.0300 (4)	
N2	0.6528 (3)	0.1678 (3)	0.40592 (14)	0.0362 (4)	
O	0.3173 (3)	0.2976 (3)	−0.16914 (12)	0.0487 (4)	
OW	0.2791 (3)	0.1012 (3)	0.42756 (18)	0.0602 (6)	
C1	0.3886 (3)	0.2802 (3)	−0.07645 (16)	0.0375 (5)	
C2	0.2788 (4)	0.2306 (4)	−0.00674 (18)	0.0475 (6)	
C3	0.3570 (3)	0.2225 (4)	0.08687 (17)	0.0447 (6)	
C4	0.5440 (3)	0.2645 (3)	0.10896 (15)	0.0319 (4)	
C5	0.6549 (3)	0.3101 (3)	0.03969 (17)	0.0395 (5)	
C6	0.5760 (4)	0.3167 (3)	−0.05438 (17)	0.0422 (5)	
C7	0.6411 (4)	0.3453 (3)	0.37993 (17)	0.0416 (5)	
C8	0.5389 (4)	0.3477 (3)	0.28063 (17)	0.0402 (5)	
C9	0.6456 (4)	0.0825 (3)	0.23609 (17)	0.0363 (5)	
C10	0.7445 (3)	0.0816 (3)	0.33578 (17)	0.0379 (5)	
HWA	0.193 (6)	0.050 (5)	0.384 (3)	0.082 (12)*	
HWB	0.227 (6)	0.161 (5)	0.454 (3)	0.080 (13)*	
H1	0.213 (5)	0.259 (5)	−0.172 (3)	0.066 (11)*	
H2	0.144 (4)	0.201 (4)	−0.019 (2)	0.055 (8)*	
H3	0.284 (4)	0.180 (4)	0.134 (2)	0.054 (8)*	
H5	0.781 (4)	0.344 (4)	0.056 (2)	0.052 (8)*	
H6	0.646 (4)	0.344 (4)	−0.102 (2)	0.052 (8)*	
H7A	0.578 (5)	0.390 (4)	0.426 (2)	0.064 (9)*	
H7B	0.769 (5)	0.410 (4)	0.385 (2)	0.054 (8)*	
H8A	0.415 (4)	0.288 (4)	0.278 (2)	0.052 (8)*	
H8B	0.531 (4)	0.449 (4)	0.264 (2)	0.051 (8)*	
H9A	0.528 (5)	0.019 (4)	0.231 (2)	0.054 (8)*	
H9B	0.718 (4)	0.045 (4)	0.191 (2)	0.045 (7)*	
H10A	0.749 (4)	−0.027 (4)	0.353 (2)	0.047 (7)*	
H10B	0.865 (4)	0.147 (4)	0.340 (2)	0.045 (7)*	
H1N	0.752 (4)	0.317 (4)	0.209 (2)	0.046 (7)*	
H2NA	0.551 (5)	0.113 (4)	0.411 (2)	0.063 (10)*	
H2NB	0.722 (4)	0.175 (4)	0.464 (2)	0.046 (8)*	

### Atomic displacement parameters (Å^2^)

**Table:**

	*U*^11^	*U*^22^	*U*^33^	*U*^12^	*U*^13^	*U*^23^
Co	0.03205 (18)	0.03383 (18)	0.03517 (19)	0.00247 (12)	0.00115 (12)	0.00040 (12)
Cl1	0.0365 (3)	0.0511 (3)	0.0436 (3)	0.0088 (2)	0.0072 (2)	−0.0033 (2)
Cl2	0.0314 (3)	0.0489 (3)	0.0448 (3)	0.0073 (2)	0.0020 (2)	−0.0056 (2)
Cl3	0.0535 (4)	0.0515 (4)	0.0533 (4)	0.0005 (3)	−0.0020 (3)	0.0208 (3)
Cl4	0.0400 (3)	0.0473 (3)	0.0682 (4)	−0.0050 (3)	0.0099 (3)	−0.0209 (3)
N1	0.0349 (9)	0.0261 (8)	0.0281 (8)	0.0025 (7)	0.0030 (7)	0.0021 (6)
N2	0.0353 (10)	0.0397 (10)	0.0316 (9)	0.0020 (8)	0.0003 (8)	0.0082 (8)
O	0.0569 (12)	0.0537 (11)	0.0325 (9)	0.0054 (9)	−0.0026 (8)	0.0072 (7)
OW	0.0417 (10)	0.0598 (13)	0.0764 (15)	0.0048 (9)	0.0080 (10)	−0.0254 (11)
C1	0.0479 (13)	0.0318 (10)	0.0322 (11)	0.0078 (9)	0.0015 (9)	0.0000 (8)
C2	0.0400 (13)	0.0566 (15)	0.0408 (13)	−0.0031 (11)	−0.0019 (10)	0.0074 (11)
C3	0.0384 (12)	0.0569 (15)	0.0347 (12)	−0.0052 (11)	0.0037 (10)	0.0090 (10)
C4	0.0378 (11)	0.0266 (9)	0.0301 (10)	0.0036 (8)	0.0014 (8)	0.0009 (7)
C5	0.0371 (12)	0.0469 (13)	0.0350 (11)	0.0074 (10)	0.0053 (9)	0.0024 (9)
C6	0.0466 (13)	0.0491 (13)	0.0322 (11)	0.0070 (11)	0.0107 (10)	0.0042 (10)
C7	0.0589 (15)	0.0334 (11)	0.0323 (11)	0.0088 (11)	0.0046 (10)	−0.0006 (9)
C8	0.0569 (15)	0.0344 (11)	0.0340 (11)	0.0193 (11)	0.0079 (10)	0.0032 (9)
C9	0.0458 (13)	0.0257 (10)	0.0375 (12)	0.0092 (9)	0.0013 (10)	0.0017 (8)
C10	0.0395 (12)	0.0332 (11)	0.0413 (12)	0.0096 (10)	−0.0003 (9)	0.0071 (9)

### Geometric parameters (Å, º)

**Table:**

Co—Cl2	2.2475 (7)	C2—C3	1.385 (3)
Co—Cl4	2.2772 (7)	C2—H2	0.99 (3)
Co—Cl1	2.2777 (7)	C3—C4	1.376 (3)
Co—Cl3	2.2868 (7)	C3—H3	0.95 (3)
N1—C4	1.484 (3)	C4—C5	1.376 (3)
N1—C9	1.500 (3)	C5—C6	1.391 (3)
N1—C8	1.506 (3)	C5—H5	0.94 (3)
N1—H1N	0.94 (3)	C6—H6	0.91 (3)
N2—C10	1.482 (3)	C7—C8	1.513 (3)
N2—C7	1.491 (3)	C7—H7A	0.94 (3)
N2—H2NA	0.83 (4)	C7—H7B	1.00 (3)
N2—H2NB	0.91 (3)	C8—H8A	0.96 (3)
O—C1	1.370 (3)	C8—H8B	0.86 (3)
O—H1	0.79 (4)	C9—C10	1.507 (3)
OW—HWA	0.87 (4)	C9—H9A	0.93 (3)
OW—HWB	0.77 (4)	C9—H9B	0.95 (3)
C1—C6	1.376 (4)	C10—H10A	0.92 (3)
C1—C2	1.382 (4)	C10—H10B	0.96 (3)
			
Cl2—Co—Cl4	111.38 (3)	C3—C4—N1	120.36 (19)
Cl2—Co—Cl1	106.87 (3)	C4—C5—C6	119.3 (2)
Cl4—Co—Cl1	113.97 (3)	C4—C5—H5	120.3 (19)
Cl2—Co—Cl3	109.60 (3)	C6—C5—H5	120.3 (19)
Cl4—Co—Cl3	108.94 (3)	C1—C6—C5	119.6 (2)
Cl1—Co—Cl3	105.87 (3)	C1—C6—H6	119.3 (19)
C4—N1—C9	111.55 (16)	C5—C6—H6	121.1 (19)
C4—N1—C8	113.32 (17)	N2—C7—C8	110.53 (19)
C9—N1—C8	110.62 (17)	N2—C7—H7A	106 (2)
C4—N1—H1N	105.0 (18)	C8—C7—H7A	111 (2)
C9—N1—H1N	106.4 (18)	N2—C7—H7B	108.3 (18)
C8—N1—H1N	109.6 (17)	C8—C7—H7B	111.9 (18)
C10—N2—C7	110.87 (18)	H7A—C7—H7B	109 (3)
C10—N2—H2NA	111 (2)	N1—C8—C7	110.1 (2)
C7—N2—H2NA	112 (2)	N1—C8—H8A	107.9 (19)
C10—N2—H2NB	109.1 (18)	C7—C8—H8A	110.1 (19)
C7—N2—H2NB	106.2 (18)	N1—C8—H8B	109 (2)
H2NA—N2—H2NB	108 (3)	C7—C8—H8B	112 (2)
C1—O—H1	105 (3)	H8A—C8—H8B	107 (3)
HWA—OW—HWB	102 (4)	N1—C9—C10	110.75 (18)
O—C1—C6	117.2 (2)	N1—C9—H9A	108.8 (19)
O—C1—C2	122.2 (2)	C10—C9—H9A	111.0 (19)
C6—C1—C2	120.6 (2)	N1—C9—H9B	103.1 (17)
C1—C2—C3	119.9 (2)	C10—C9—H9B	109.7 (17)
C1—C2—H2	123.5 (18)	H9A—C9—H9B	113 (2)
C3—C2—H2	116.6 (18)	N2—C10—C9	110.96 (19)
C4—C3—C2	119.1 (2)	N2—C10—H10A	108.8 (18)
C4—C3—H3	120.2 (18)	C9—C10—H10A	110.8 (18)
C2—C3—H3	120.5 (18)	N2—C10—H10B	104.4 (17)
C5—C4—C3	121.4 (2)	C9—C10—H10B	110.3 (17)
C5—C4—N1	118.25 (19)	H10A—C10—H10B	111 (2)

### Hydrogen-bond geometry (Å, º)

**Table:**

*D*—H···*A*	*D*—H	H···*A*	*D*···*A*	*D*—H···*A*
O*W*—H*WA*···Cl3^i^	0.87 (4)	2.59 (4)	3.381 (3)	150 (4)
O*W*—H*WB*···Cl1^ii^	0.78 (4)	2.51 (4)	3.264 (3)	164 (4)
O—H1···Cl3^iii^	0.79 (4)	2.41 (4)	3.177 (3)	165 (4)
N1—H1*N*···Cl4^iv^	0.94 (3)	2.24 (3)	3.171 (2)	170 (2)
N2—H2*NA*···O*W*	0.83 (4)	2.06 (4)	2.807 (2)	151 (3)
N2—H2*NB*···Cl1^v^	0.91 (3)	2.47 (3)	3.267 (2)	147 (2)
N2—H2*NB*···Cl2^v^	0.91 (3)	2.81 (3)	3.324 (2)	117 (2)

Symmetry codes: (i) *x*, *y*−1, *z*; (ii) −*x*, −*y*+1, −*z*+1; (iii) −*x*, −*y*+1, −*z*; (iv) *x*+1, *y*, *z*; (v) −*x*+1, −*y*+1, −*z*+1.
